# Multi-functional, multicompartmental hyaluronan-binding protein 1 (HABP1/p32/gC1qR): implication in cancer progression and metastasis

**DOI:** 10.18632/oncotarget.24082

**Published:** 2018-01-09

**Authors:** Paramita Saha, Kasturi Datta

**Affiliations:** ^1^ Biochemistry and Toxicology Laboratory, School of Environmental Sciences, Jawaharlal Nehru University, New Delhi 110067, India

**Keywords:** hyaluronan-binding protein 1 (HABP1/p32/gC1qR), structural flexibility, hyaluronan (HA), tumor-biomarker, serine-arginine rich splicing factor 1 (SRSF1)

## Abstract

Cancer is a complex, multi-factorial, multi-stage disease and a global threat to human health. Early detection of nature and stage of cancer is highly crucial for disease management. Recent studies have proved beyond any doubt about the involvement of the ubiquitous, myriad ligand binding, multi-functional human protein, hyaluronan-binding protein 1 (HABP1), which is identical to the splicing factor associated protein (p32) and the receptor of the globular head of the complement component (gC1qR) in tumorigenesis and cancer metastasis. Simultaneously three laboratories have discovered and named this protein separately as mentioned. Subsequently, different scientists have worked on the distinct functions in cellular processes ranging from immunological response, splicing mechanism, sperm-oocyte interactions, cell cycle regulation to cancer and have concentrated in their respective area of interest, referring it as either p32 or gC1qR or HABP1. HABP1 overexpression has been reported in almost all the tissue-specific forms of cancer and correlated with stage and poor prognosis in patients. In order to tackle this deadly disease and for therapeutic intervention, it is imperative to focus on all the regulatory aspects of this protein. Hence, this work is an attempt to combine an assortment of information on this protein to have an overview, which suggests its use as a diagnostic marker for cancer. The knowledge might assist in the designing of drugs for therapeutic intervention of HABP1/p32/gC1qR regulated specific ligand mediated pathways in cancer.

## INTRODUCTION

Extracellular matrix (ECM) is not only crucial for the maintenance of tissue architecture, but it essentially regulates gene expression under normal condition as well as during pathophysiological conditions, which include tumor progression and metastasis. The crosstalk between cancerous cells with their surrounding ECM creates a favorable microenvironment or niche and enables tumor progression and metastasis. A variety of the ECM effects are arbitrated by dynamic interactions between them and their binding partners followed by cellular signaling. Among the numerous ECM components, hyaluronan (HA), a mucopolysaccharide, is reported to regulate several processes like embryogenesis, immunological reactions, cell differentiation and epithelial mesenchymal transition [1]. For the past few decades, immense importance has been given to the role of HA in cancer progression. A family of proteins termed as ‘hyaladherins’ having link protein B-(X)_7_-B motif, binds specifically to HA, which reportedly mediate the diverse ligand multitude cellular activities [2] and also regulate tumor formation and progression of cancer [3, 4].

## THE UNEARTHING OF HABP1

An important hyaladherin, hyaluronan-binding protein 1 (HABP1) was purified using HA affinity chromatography and identified as a glycoprotein containing sialic acid by *D'Souza and Datta* in 1985. This protein was initially referred to as hyaluronectin [5]. Amino acid composition studies revealed that HABP1 is rich in glycine and glutamic acid and it is distinct from other HA binding proteins such as fibronectin, link protein and aggrecan [6, 7]. After establishing its uniqueness, the insoluble extract of rat kidney tissue was fractionated and this HA binding protein was purified using HA affinity chromatography to homogeneity. The molecular weight of the isolated native protein was 68 kDa, consisting of two sub-units of 34 kDa on SDS-PAGE [8]. Highest specific affinity of this protein towards HA (K_d_ 1X 10^−9^M) amongst all glycosaminoglycans (GAGs) along with its interaction with fibronectin, laminin and collagen has been confirmed. This protein has been found to be secreted in the medium and its localization in fibroblast confirmed its presence on the cell surface [9]. It is reported to be present in almost all tissues types except red blood cells (RBCs) and has been shown to be hyperphosphorylated by HA in lymphocytes [9]. Simultaneously, its adhesive nature and regulatory role in solid tumor formation [10], as well as enhanced phosphorylation in transformed cells is also reported [11]. Moreover, phosphorylation of this protein upon PMA stimulation, calyculin and Ca^2+^ ionophore has been found to be regulated by PI3-kinase; which indicated its probable role in cellular signaling [12]. This supposition has been further strengthened from the observation of nuclear translocation of HABP1, upon PMA stimulation which can be blocked by the introduction of MAP kinase inhibitor, PD98059; implying HABP1 to be an endogenous MAP-kinase substrate [13]. In continuation, it was relevant to study the regulatory role of HABP1 in reproduction since, HA is an important molecule in reproductive fluids. Specific function of HABP1 in sperm motility [14–17], sperm oocyte interaction [18] and in folliculogenesis [19–21] has been well documented by our laboratory.

Subsequent to the establishment of the probable involvement of HABP1 in diverse regulatory processes related to HA, our laboratory was involved in identifying the gene encoding this protein, in order to study its regulation and its functional relevance.

### Molecular cloning and chromosomal localization: identity with p32 and gC1qR

Antibodies raised against HABP1 have been used to identify the gene from λgt expression library of human fibroblast and was subsequently sequenced. The amino acid sequence of the gene identified was in complete agreement with thirteen polypeptides derived from the HABP1 protein, thus confirming its identity. The recombinant protein has been purified using HA affinity chromatography after overexpressing it in *E. coli* and its immunological identity and similar HA affinity has also been validated [22].

The cDNA sequence of 34 kDa HABP completely matched with the cDNA sequence of a protein named p32 [22], which happened to have been co-purified with the splicing factor SF2 [23, 24]; (Genbank ID L04636 and M69039). The sequence is even identical to that of the human receptor for the globular head of the complement factor 1q, gC1qR [25], which was already reported by *Ghebrehiwet et al*, 1994 (Genbank ID X75913) [26]. This 34 kDa HABP was given the accession ID: 9786126 and named as HABP1 by the Hugo Nomenclature Committee of GDB. Therefore, this protein will be referred here as HABP1/p32/gC1qR. Later on, as a policy of HUGO, the official name of this protein was considered as gC1qR, but referred to as either p32 or HABP1 or gC1qR in various reports in the literature. Therefore, it is immensely necessary to compile all the published literature to have an overview of the multitude of functions of HABP1 in the diverse biological processes.

It was initially presumed that p32 cDNA does not contain any conventional ATG (Met) start codon but initiates with a CTG (Leu) codon [24]. Further studies though revealed that the entire p32 cDNA extends beyond the 5' end of the cDNA as reported by *Krainer et al* (1991) and ATG is indeed the start codon [23]. However, the recombinant protein produced from cells infected with *Vaccinia* virus harboring the p32 cDNA starting with the ATG start codon had an N-terminal amino acid sequence identical to that reported by *Krainer et al* [24]. This led to the discovery of the post-translational processing of the 282 amino acid containing pro-protein of HABP1 into generation of the mature protein of 209 amino acids, by the removal of the initial 73 amino acids [23]. Out of the 73 amino acid residues, the first 13 residues forms a leader peptide, while the next 60 residues preceding the mature protein is comprised of a long hydrophobic stretch containing five cysteine residues. The leader peptide contains the mitochondrial targeting signal sequence [27, 28]. The calculated molecular weight of the mature protein corresponds to 23.7 kDa but interestingly, it migrates at 34 kDa on SDS-PAGE due to the higher ratio of polar to hydrophobic amino acid residues. The calculated pI of 4.15 for the mature protein suggests it to be acidic in nature [29].

Search for the probable motif enabled us to identify the minimal motif required for the binding of HABP1 to HA, which is referred to as the B-(X)_7_-B motif; where B is either R or K and X_7_ is a stretch of 7 non-acidic amino acid residues in between [30]. The motif ^119^KLVRKVAGEK^128^ represents the HA binding motif in mature HABP1. This motif however contains an extra glutamic acid residue, but the crystal structure reveals that the accessibility of glutamic acid is very low, as it forms a salt-bridge with Arg^246^. This effectively generates the B-(X)_7_-B motif at the binding site for HA, confirming HABP1 as a new member of hyaladherins [29]. Though the protein does not possess a consensus bipartite sequence, which would direct it to the nucleus, two basic amino acid rich putative nuclear localization signals (NLS) ^94^RKIQKHK^100^ and ^118^AKLVRK^123^ overlapping with the HA binding motif, have been identified in HABP1 which explains the localization of the protein in the nucleus [31].

Using fluorescence *in situ* hybridization (FISH) analysis, the HABP1 gene was mapped at chromosome 17p12-p13. This chromosomal localization shows 99.5% similarity (from base 928 to base 1163) with STS WI-9242, an STS flanking marker of human chromosome 17 [32]. The existence of HABP1 pseudogenes in humans was revealed upon genomic search with HABP1 cDNA. These pseudogenes are located in chromosomes 21, 15, 11 and 4 varying in length and similarity to parental cDNA sequence [33]. Interestingly, all four pseudogene like sequence of HABP1 has been detected in *Methanosarcina barkeri*, an ancient life form. This sequence has 44.8% homology with human HABP1 cDNA and 45.3% homology with HABP1 pseudogene in human chromosome 21 [34].

### Homologues of HABP1

HABP1 is a conserved eukaryotic protein ubiquitously present from yeast to mammals. The homologues of HABP1 have been reported from mouse, rat, chicken, *C. elegans*, *Saccharomyces cerevisiae* and *Trypanosoma brucei*. A common feature in all HABP1 homologues is the conserved nature of polar amino acid residues. In almost all the homologues, ratio of positively charged amino acid to negatively charged residues is same and is always less than one [34].

Human HABP1/p32 is shown to exhibit 53% similarity and 26% identity to its yeast homologue Mam33p found in *S. cerevisiae.* Like HABP1/gC1qR/p32, it is synthesized as a precursor with an N-terminal mitochondrial targeting sequence, which gets processed upon translocation to the mitochondrial matrix. In the mitochondrial matrix, Mam33p gets assembled into a homo-oligomeric complex; which leads to binding with the sorting signal of cytochrome b2. This ultimately guides this protein into the inter-membrane space. Also, they have shown similar biochemical features like an isoelectric point at around 4 and abnormal migration on SDS-PAGE probably due to the prevalence of acidic residues [35]. Recently, Mam33 has been shown as an activator of translation of mitochondrially encoded Cox1, a subunit of cytochrome C oxidase. Mam33 is also critical for cells to adapt easily from fermentation to respiration mode [36].

The mouse homologue of HABP1 gene is mapped and reported to be located on chromosome 11 [37]. It spans approximately 6 kb of the mouse genome and comprises of six exons separated by five introns. Exon 1 containing a long stretch of 70 amino acid residues is reported to encode for the putative mitochondrial localization signal peptide. The first four amino acid residues are found in the mature gC1qBP. By comparing the cDNA-derived amino acid sequences, the degree of identity is mapped to 89.9% between human and rodent gC1qBP and 97.6% between the rat and mouse gC1qBP. The amino acid differences are mostly confined to the N-terminus while all the three potential glycosylation sites are found to be conserved [37].

The chicken homologue is a 38-kDa protein on SDS-PAGE and it is almost 90% homologous with its human counterpart. It has been shown under *in vitro* conditions that it can bind to de-phosphorylated myosin and assists in myosin assembly to form filament [38]. However, sequence search has established its homologues in numerous other species, though experimental studies on these proteins have not been carried out in details. These include counterparts in *Cercopithecus aethiops*, *Drosophila melanogaster*, *Xenopus laevis*, *Caenorhabditis elegans*, *Gallus gallus* and a putative protein in *Schizosaccharomyces pombe* [34]. A comparative sequence analysis of HABP1 homologues across species showed a conservation of the C-terminal region. The glutamic acid residue Glu-127, located in the HA-binding motif ^119^KLVRKVAGEK^128^ is an invariant residue, being perfectly conserved across species. The basic residues of the HA binding motif Lys/Arg-119, Lys/Arg-122, Lys/Arg-123 are also fairly conserved across species. The lone cysteine residue, cys-186 is conserved only in mammals in comparison to other eukaryotic species [22, 29, 39]. A gene encoding mrb1, required for mating and to form dikaryotic hyphae in *Ustilago maydis*, has been found to have significant similarity with mitochondrial HABP1/p32 protein [40].

### Structural plasticity of HABP1 regulating ligand affinity and cellular functions

Determination of the crystal structure of HABP1 at a resolution of 2.25 Ǻ reveals that, HABP1 monomer adopts a novel fold with seven consecutive anti-parallel β-strands flanked by one N-terminal (αA) and two C-terminal (αB and αC) α-helices. The seven consecutive β-strands, designated β1 to β7, forms a highly twisted anti-parallel β-sheet, with β1 nearly perpendicular to β7. All the three helices are located on the same side of the β-sheet. The coiled coil region of αA and αC forms extensive intermolecular contacts. The N-terminal helix, αA does not contact the β-sheet within the monomer, but forms an anti-parallel coiled-coil with the anti-parallel αB of an adjacent monomer and the C-terminal region of αC packs against the back of the β-sheet. These intermolecular interactions are mostly hydrophobic in nature [39]. A doughnut-shaped quaternary structure can be formed by three of these monomers, with an unusually asymmetric charge distribution on the surface. All three subunits have very similar conformations and apparently distinct domains have not been observed in monomeric HABP1 [39].

The HA-binding motif has been mapped to amino acids 119-128 of HABP1, which corresponds to a region between β2 and β3, with a loop in between. HABP1 does not have the canonical HA-binding motif, as the presence of Glu-127 disrupts the B-(X)_7_-B motif. This residue though conserved across species, forms a salt-bridge with Arg-246, another conserved residue. This renders the glutamic acid inaccessible to the surface and generates the typical HA-binding motif [29, 39]. Later on, we structurally modelled HABP1 and HA interaction which demonstrated that, HA binds strongly with HABP1, via hydrophobic as well as hydrogen bond interactions [41]. Moreover, Arg-122 of the HA binding motif plays a key role in the first linkage making hydrogen bond with glucuronic moiety of the sugar and the secondary alcohol group of the sugar succeeding the linkage. Motif residue of Arg-122 and Ala-125 also participate in hydrophobic interaction with the ligand. In summary, the overall architecture of the trimer can be visualized as if the β-sheet forms a hyperboloid shaped spool with the α-helices wrapped around it [41].

The trimeric HABP1/p32/gC1qR has a potential to give rise to a functionally relevant dimer of trimers in an oxidative environment through the only cysteine Cys^186^ present in each protomer [29]. Though the crystal structure shows that the cysteine is completely buried and is not available for dimerization yet; this cysteine has been observed to mediate dimerization of trimeric HABP1/p32/gC1qR, at near physiological conditions or in relatively low ionic environment. The three-dimensional structural arrangement in low ionic environment near neutral to alkaline pH differs from the crystallographically determined structure obtained in high ionic and reducing environment [39]. In addition, it was observed that the disulfide-mediated dimer of trimer shows significantly higher binding towards HA as compared to trimeric HABP1/p32/gC1qR as well as other reported ligands such as mannosylated albumin (DMA) [42] and gC1q [26]. In addition to the different oligomeric forms of HABP1 under various conditions, we further reported its structural transition, induced by the ionic environment around the molecule which regulates the affinity of this protein for different ligands [43]. At low ionic environment, HABP1 exists in an expanded molten globule like state, which attains a globular conformation above 50 mM salt concentration, but with higher salt concentration (150 mM-1M) a compact structure appears. Such structural plasticity is shown to regulate the affinity of different ligands since at 10 mM salt concentration HA does not have any affinity, but other ligands e.g. C1q and clustered mannose appreciably binds to HABP1. Even the effect of ionic strength and pH affect the thermodynamic stability of HABP1 [44]. The characteristic feature of HABP1 such as anomalous migration in SDS-PAGE, high vulnerability to proteolysis and lack of secondary structure at low ionic concentration, indicate HABP1 can be an intrinsically unstructured protein. This hypothesis was confirmed by amino acid frequencies compared with that of ordered and intrinsically unstructured protein [44]. Multifunctional, multi-ligand protein HABP1 is reported to have assorted biological activities and structural flexibility is the key to such diverse functions. The presence of molten globule like structure either in absence or very low concentration of salt seems to be responsible for its molecular chaperone like activity in interacting with protein kinase C isoforms or mitochondrial p14ARF [45]. The unique feature of this protein, being a mitochondrial protein is that it shows its presence in the other cellular compartments e.g. cell surface, cytoplasm and nucleus, having asymmetric changes. Trimeric doughnut shaped, crystal structure of this protein suggests the tunneling or trafficking pathway connecting the nucleus, mitochondria and cytoplasm and the export pathway to cell surface since it is reported to interact with many other cellular proteins having WD-Family characteristics. These observations indicate that HABP1/p32/gC1qR has a substantial degree of structural flexibility, which allows the reorganization of the oligomeric assembly [43]. The crystal structure of HABP1/p32 has shed light on many newer aspects of HABP1 function by predicting its possible involvement in numerous cellular processes and helped in understanding its functional diversity and sub-cellular localization pattern.

### Diverse sub-cellular localization and myriad ligands

Over a decade, myriad proteins have been reported to bind to HABP1. These studies suggest that HABP1 displays the ability to bind with a number of apparently unrelated proteins, which seem to have key functions in different cellular processes at different sub-cellular locations. The pleiotropic nature of HABP1 is attributed to its binding affinities with so many different genres of ligands. An overview of its different ligands which make its role in cellular signaling and apoptosis more apparent is represented in Table 1.

**Table 1:** Reported subcellular localization HABP1 and respective interacting proteins

	Function	Reference
**Interacting Proteins:** ***CELL SURFACE***		
**gC1q**	It prevents the immune complexes from binding to the globular heads of C1q	[26]
**cC1qR/Calreticulin (CR)**	CR has been shown to be involved in a nuclear export pathway of the glucocorticoid receptor	[46, 47]
**Fibrinogen**	It is speculated to aid in the modulation of fibrin formation during injuries and inflammation	[48]
**Tumor homing peptide, Lyp-1**	It specifically recognizes an epitope in tumor lymphatics/tumor cells, also acts as a marker in certain cancers	[49]
**Matrix-metalloproteinase, MT1-MMP**	They are likely to be involved in the machanisms regulating the presentation of the protease at the tumor cell surface	[50]
**Lethal giant larvae (Lgl)**	p32 regulates cell polarity by forming a complex with mammalian Lgl2 and atypical protein kinase C (aPKC) and enhances aPKC activity	[51]
**Interacting proteins: *NUCLEUS***		
**Pre mRNA splicing factor (SF2/ASF)**	It controls RNA splicing by sequestering an essential RNA splicing factor into an inhibitory complex	[52]
**Lamin B Receptor [p58]**	It acts as a linker between the nuclear membrane and intranuclear spliceosomal substructures	[53]
**Transcription factor 11B**	It has been suggested to function as a cellular co-activator, bridging Tat to the general transcriptional machinery	[54]
**CCAAT Binding Factor (CBF/NF-y)**	Mutations in the CCAAT motifs prevent CBF binding, decreasing the transcriptional activity	[55]
**human forkhead box C1 (FOXC1)**	Human p32 is a FOXC1-interacting protein that regulates FOXC1 transcriptional activity in ocular cells	[56]
**CDC2L5, a Cdk-Like Kinase**	p32 interacts with CDC2L5, a Cdk-Like Kinase containing RS domain, and affects splicing *in vivo*	[57]
**Interacting proteins: *CYTOPLASM:***		
**Short Mitochondrial ARF (smARF)**	smARF is a inducer of type 2 autophagic cell death and its association with p32 specifically regulates the expression of autophagy	[58, 59]
**Alpha 1B-adrenergic receptor**	The expression level and cellular localization of α1β-AR is governed through its interaction with HABP1/gC1qR	[60]
**Cytochrome b2**	Mam33p, yeast homologue of HABP1 is not essential for cytb2 sorting signal that directs the protein to the intermembrane space	[35]
**Protein kinase C μ**	HABP1/p32 is a part of an intracellular receptor that restricts PKCμ at an intracellular compartment such as mitochondria and modulates its kinase activity	[45]
**g-Amino Butyric Acid Receptor (GABAA)**	The presence of functionally relevant Ser-410 within the interacting site suggests a modulatory role of gC1qR either in biosynthesis or the mature receptor	[61]
**Hrk/DP5**	p32 may be a key molecule that links Hrk to mitochondria and is critically involved in the regulation of HRK mediated apoptosis	[62]
**cdc25**	HABP1 induces morphological changes like elongation, multinucleation and aberrant cell septum formation in *Schizosaccharomyces pombe* and modulates the cell cycle by interacting with proteins like CDC25 through its N-terminal a-helix	[63]
**Mcl-1**	Mcl-1 binds with p32 and positively regulates mitochondrial Ca^2+^ uptake and apoptosis	[64]
**RECQ4**	p32 promotes the nuclear localization of RECQ4 by suppressing its transport to mitochondria	[65]
**c-Myc**	Myc promotes the expression of p32, which is required to maintain sufficient respiratory capacity to sustain glutamine metabolism in Myc transformed cells	[66]
**Parkin**	p32 is a novel interacting partner of parkin in the brain. It regulates mitochondrial morphology and dynamics by promoting parkin degradation through autophagy	[67]

### HABP1 in inflammation, infection and immune recognition

The cellular activities of HA is diverse in nature including cell adhesion, cell migration, T-cell activation, B-cell maturation, thus regulating immunity of an organism. Our lab has earlier demonstrated the presence of HABP1 on lymphocytes and HA induced aggregation in association with hyperphosphorylation and IP3 formation which is inhibited by anti-HABP1 antibody [9]. Even the role of HABP1 was anticipated in visceral Leishmaniasis. Studies revealed elevated levels of HABP1 in spleen, liver and serum of *Leishmania donovani* infected hamster, in an *in vitro* model of macrophage cell line, J774.G8 and also in the serum of Kala-azar patients [68], indicating a role of HABP1 in infection and immunity. Our report on the identity of HABP1 with gC1qR [25] and its interaction with the globular head of the complement subcomponent1 (gC1q) [26], its involvement in immunity became apparent. The complement cascade has been well studied and the activity of C1q is not only limited to the recognition and initiation of the classical complement activation pathway, but it can also mediate immune effector functions, influencing inflammation and immunity directly [69]. These responses are mediated by cell surface adapter molecules cC1qR and gC1qR/HABP1. cC1qR is a high affinity calcium binding protein, which binds to the collagen tail of C1q, whereas, gC1qR binds at the globular head of C1q and mediate several biochemical and cellular activities [70]. The elegant work of *Ghebrehiwet* group from Stony Brook confirmed the affinity of 33kDa gC1qR/HABP1 isolated from Raji B cell towards C1q to be approximately 50–100 nM under physiological ionic strength (150mM NaCl), while it is higher at sub-physiological (90 mM NaCl) conditions [26]. In cells expressing both gC1qR and cC1qR (calreticulin), C1q interaction mediates a wide range of cellular activities which includes inositol-tri-phosphate (IP3) production, pro-coagulatant activity on platelets and mast cell chemotaxis [70, 71]. HABP1/gC1qR - gC1q interaction reportedly inhibits complement activation thus, preventing the immune complexes from binding to the globular heads of C1q. First 18 amino acid residues from N-terminus of mature gC1q-R/HABP1 (76-93) have been reported to contain a major binding site for C1q [26, 71]. HABP1/gC1qR has also been shown to bind specifically to the heparin-binding multimeric form of vitronectin and the ternary complex vitronectin-thrombin-antithrombin complex. Thus, HABP1 acts as a novel vitronectin-binding protein that may participate in the clearance of vitronectin-containing complexes or opsonized particles or cooperate with vitronectin in the inhibition of complement-mediated cytolysis [72]. It has been reported that HABP1 binds to several serum proteins, and thus plays a key role in blood clotting [73] and fibrin polymerisation [48]. gC1qR/HABP1 can also act as a receptor and interact with several proinflammatory ligands e.g. high molecular weight kininogen (HK), factor XII. HK and Factor XII compete to bind to HABP1/gC1qR suggesting that an endothelial binding protein mediates the assembly of critical components of the kinin-generating pathway on the surface of endothelial cells, thereby linking the early events of kinin formation and complement activation [27, 74–76] affecting plasma-kinin forming system. Ability of HABP1 to recognize and bind to plasma proteins (e.g.C1q and HK) and a wide array of viral and bacterial antigens not only leads to generation of pro-inflammatory byproducts and kinin/kallikrein systems but also acts as a route of host-pathogenic microbial reactions [69, 76].

There are several reports linking HABP1/p32/gC1qR with pathogenic infection via its binding to several parasitic, viral and bacterial proteins [76, 77]. In addition to mitochondria and cell surface, HABP1 has been shown to be localized mainly to the nucleus and cytosol by immunolocalization studies using tagged HABP1 [78]. Interaction of HABP1 with viral proteins also targets it to the nucleus [31, 79]. Interaction between Epstein Barr virus nuclear antigen-1 (EBNA-1) and p32/HABP1/gC1qR has been suggested to promote ori-P dependent DNA replication in the EBV infected cell during the S-phase of the cell cycle [80]. Human Immunodeficiency Virus Type-1 Rev and Tat binding with HABP1 reportedly has role in HIV replication [54, 81]. Even the recent report on respiratory syncytial virus (RSV) suggests the involvement of HABP1/gC1qR in the replication and infectious viral production. p32 knockdown using siRNA led to reduction of viral replication and production along with a clear change in mitochondrial organization [82]. gC1qR/HABP1 has also been implicated in the pathogenesis of hepatitis C virus (HCV) associated mixed cryoglobulinemia (MC) and vascular damage. Nonenveloped HCV core protein circulating in the blood reportedly interacts with gC1qR/HABP1 and MC patients have elevated levels of gC1qR in the blood. Cryoglobulins which are cold-precipitable Ig make patients susceptible to the development of vascular, renal and neurological lesions and HCV positive patients are prone towards the occurrence of malignant lymphoproliferative disorder [83].

In platelets, binding of gC1qR/HABP1 with the virulence factor, proteinA of *Staphylococcus aureus* has been reported as a mechanism for bacterial cell adhesion and localization to sites of vascular injury and thrombosis [84]. It also acts as a receptor for internalization of the bacteria, *Listeria monocytogenes* by interacting with the bacterial protein Internalin IA (InIA) [85].

It has been demonstrated by our laboratory for the first time that, *Plasmodium falciparum* infected RBCs (iRBCs) use the 32-kDa human protein gC1qR/HABP1/p32 as a receptor to bind to human endothelial cells, including brain microvascular endothelial cells. It was also observed that *P. falciparum* iRBCs can bind to gC1qR/HABP1/p32 on platelets to form clumps. Thus, gC1qR/HABP1/p32 was identified as a novel host receptor that is used for both adhesion to vascular endothelium and platelet mediated clumping in severe cases of malaria [86]. Further work has been carried out on *P. falciparum* clinical isolates from Mozambican children suffering from severe malaria and uncomplicated malaria. The study indicated the cytoadherence of *P. falciparum* to gC1qR to be one of the major virulent factors, which along with platelet mediated clumping and rosetting is involved in the pathogenesis and severity of malarial infection [87, 88]. Participation of HABP1 in regulating bacterial invasion is apparent, since the activity of *Staphylococcus pneumoniae* hyaluronate lyase (SpnHL), which is considered as the spread factor; is inhibited by HABP1 in a concentration dependent manner with IC_50_ value of 20 μM. Kinetic analysis and structural docking experiment of SpnHL with HABP1 confirm it as a competitive inhibitor against SpnHL mediated mammalian HA degradation. *In silico* analysis revealed that HABP1 binds and blocks a large number of crucial group of residues in the active cleft of SpnHL [41]. Dissimilarity in the sequence of the bacterial and mammalian hyaluronidases contrary to the high degree of sequence homology associated with the active cleft of majority of bacterial hyaluronate lyases, makes them vulnerable to similar inhibition by HABP1. This discovery of inhibition of SpnHL competitively by HABP1 offers an opportunity for therapeutic intervention and designing a peptide or identifying a synthetic small molecule drug against bacterial infection [41, 77].

Upon retrospection of majority of the reports here, it is evident that HABP1/gC1qR induces EBV and HIV replication and augmented levels of HABP1 can be co-related with severity of protozoal (e.g. *P. falciparum*) and viral (e.g. HCV) infections. During pathogen-host interactions, HABP1/gC1qR not only interacts with proinflammatory ligands but can also act as a vehicle for pathogenesis [76]. Recent studies have focused on the critical role of inflammation in tumor progression. This has been conceptualized since several cancers arise from sites of infection, chronic irritation and inflammation. Tumor microenvironment is hugely governed and influenced by inflammatory cells; and their participation in the neoplastic process is very vital [89]. Thus, it can be speculated from all the above evidences that HABP1/gC1qR might have a part to play in the process of inflammation associated neoplasia.

### Mitochondrial dynamics: regulation by HABP1

Cellular mitochondrial dynamics is being reported to regulate cell survival or death. This signaling process is enormously crucial and carried out by different mitochondrial proteins. HABP1, an evolutionarily conserved protein from yeast to human, exists predominantly in mitochondria [28], though its localization in other cellular compartments in a variety of cell lines under diverse conditions has been observed [13, 31, 49]. Having mitochondrial signal sequence in the precursor form of HABP1 [28], the protein is also expected to be localized in the mitochondria and to regulate the mitochondrial activities e.g. oxidative phosphorylation, maintenance of mitochondrial structure and other related activities. Homozygous disruption of p32 gene in mice leads to mid-gestation lethality along with defects in OXPHOS because of defective protein synthesis encoded by mitochondrial DNA. From this observation, it has been hypothesized that p32/HABP1 is one crucial RNA binding factor for translation of mitochondrial proteins and is thus considered as indispensable for fetal development [90]. Stable overexpression of HABP1 in normal fibroblasts reportedly leads to growth retardation, ROS generation, autophagic vacuole formation, mitochondrial dysfunction, mitochondrial Ca^2+^ uptake and drop in mitochondrial membrane potential and subsequently induces apoptosis [91–93]. These alterations are associated with changes which occur during degeneration and aging hence, the level of HABP1 is highly crucial for the balance. Interestingly, upon supplementation of polymeric HA, significant decline in ROS induced autophagic vacuolation has been observed in HABP1 overexpressing fibroblasts [93]. The mitochondrial protein is expected to mediate in the process by interacting with other proteins, e.g. mainly with tumor suppressor, ARF [59, 94] and with Mcl-1, a major anti-apoptotic Bcl2 family protein [64]. The tumor suppressor ARF carries several cellular activities depending on its subcellular localization. Nucleolar localized ARF interacts with Mdm2, inhibiting cell cycle progression. However, in mitochondria, it acts as an autophagic modulator and proapoptotic protein. Translation initiation of ARF mRNA from an internal methionine codon, producing a short ARF protein lacking the N-terminal 47 amino acids; does not have the capacity to bind with Mdm2 [94]. This short form of ARF devoid of localization signal gets localized in the mitochondria and is named as short mitochondrial ARF or smARF. smARF is a very unstable protein due to proteasomal degradation. Enhanced expression of smARF reduces the mitochondrial membrane potential, leading to induction of autophagy; instead of cytochrome C release or caspase activation [58, 59]. Mass spectrometry based approach indicated, mitochondrial ARF to interact with the anti-apoptotic protein, Bcl-XL which otherwise protects cells from autophagy by inhibiting Beclin 1 activity. Autophagic vacuole formation along with higher expression of HABP1 and Beclin 1 is reported to occur initially, which is followed by growth arrest at G1-S phase and apoptosis induction [95, 96]. The coincidence of apoptotic induction upon cisplatin treatment in HeLa cells with upregulation of HABP1 also suggests its probable involvement in apoptosis [97].

There are several apoptotic stimuli which can initiate the process of pore formation in mitochondria and release intermembrane protein, cytochrome C in cytosol in order to commence the caspase cascade. One such apoptosis trigger is Ca^2+^ mobilization from ER to mitochondria. The Bcl-2 protein family pro-apoptotic members e.g. Bax and Bak or the ‘BH3 domain only’ proteins (e.g. Bid, Bim, Bad, Puma etc.) plays a part in the triggering process; while antiapoptotic Bcl-2 protein, Mcl-1 inhibits apoptosis by interacting with pro-apoptotic members [64]. Mcl-1 has also been reported to hinder mitochondrial Ca^2+^ uptake, thereby inhibiting apoptosis. Recently, HABP1/p32 has been revealed as a novel binding partner and antagonist of Mcl-1, positively regulating mitochondrial Ca^2+^ uptake and apoptosis in HeLa cells. Having a doughnut shaped crystal structure, with a sizeable central pore; p32 is suggested to regulate intra-mitochondrial divalent Ca^2+^ levels. Thus, HABP1 can be a direct regulator of Ca^2+^ uptake since overexpression of p32 upregulates the mitochondrial Ca^2+^ influx which can be reduced by HABP1 siRNA. Thus, it may be speculated that binding of HABP1 to Mcl-1 may suppress the apoptotic progression preventing the elevation of mitochondrial Ca^2+^ uptake [64].

Earlier observation on another Bcl-2 homology domain BH3, Hrk is known to play a critical role in mitochondrial apoptosis. HABP1 is shown to bind and co-immunoprecipitated with ‘Hrk’, BH3 domain protein through the conserved C-terminal region of p32; suggesting that HABP1 could be a key regulator that links Hrk to mitochondria playing an important role in Hrk induced apoptosis [62]. Interaction of HABP1 and the neuro-protective protein, parkin has been reported in the regulation of mitochondrial morphology and dynamics. Deregulated parkin leads to the neurodegenerative Parkinson's disease. p32 interacts with parkin in the brain and maintains the parkin protein level through autophagic degradation. It has also been reported to regulate mitochondrial movement having an implication in neurodegeneration [67].

### HABP1 and SRSF1 regulated splicing

In the search for other functions of HABP1, related to its probable regulatory role in metabolic alteration in cancer cells, we revisited the earlier report of HABP1/p32/gC1qR being co-purified during cloning and purification of splicing factor ASF/SF2, from nuclear extracts of HeLa cells [24]. SF2/ASF (renamed now as SRSF1) is the first identified member of serine arginine (SR) rich protein family and has been characterized as key regulator of constitutive pre – mRNA splicing as well as of alternate splicing. Multiple mRNA transcripts are generated in almost 95% human genes, by the process of alternate splicing. It involves the differential inclusion of exons or part of it. Several SR proteins including SRSF1 have been reported to shuttle continuously between nucleus and cytoplasm [98]. The activity and subcellular localization of SRSF1 is governed by the various post-translational modifications (PTMs). Aforementioned PTMs of SRSF1 includes extensive phosphorylation of the serine residues in various RS domains by nuclear Clk/Sty kinases or cytoplasmic SRPK kinases (Serine Arginine Protein Kinase) and dephosphorylation by phosphatases etc. The transitional intermediate phosphorylated forms of SRSF1 manipulate its interactions with other proteins and RNA binding. A hypophosphorylated form of SRSF1 generated upon cytosolic phosphorylation of 12 Ser residues at the N-terminal RS domain gets translocated to the nucleus and accumulates in nuclear speckles. This hypophosphorylated form gets hyper-phosphorylated at Ser residues by Clk/Sty kinases which propels it to the transcription active sites to induce splicing. During splicing it is restored back to the hypophosphorylated form and resumes its post-splicing function along with its nuclear export bound to spliced mRNA. Hence, RS domain of SRSF1 is crucial for nuclear-cytoplasmic shuttling and its subnuclear localization [99].

The phosphorylation of ASF/SF2, a pre-requirement for stable RNA binding and protein-protein interaction during spliceosome formation has been shown to be inhibited by p32/HABP1. HABP1 has been defined as a modular protein altering SRSF1 activity by forming an inactive complex. It has been reported that HABP1/p32/gC1qR interacts with ASF/SF2 and SRp30c, another member of the SR protein family, and plays a major role in the inhibition of ASF/SF2 interaction with RNA, but p32 does not block SRp30c function [52]. Therefore, it has been postulated that p32 can regulate RNA binding and phosphorylation of ASF/SF2. The report placed HABP1/p32/gC1qR in the group of proteins which control RNA splicing by sequestering an essential RNA splicing factor into an inhibitory complex [52]. Moreover, HABP1 regulates splicing *in vivo* by interacting with the RS domain of a Cdk-like kinase CDC2L5 [57]. Another RS motif phosphorylating kinase, lamin B receptor (LBR) kinase has shown similar substrate specificities as SRPK1. SRPK1 recognizes both LBR and ASF/SF2/SRSF1 [100]. HABP1 reportedly binds to the integral protein of the inner nuclear membrane i.e. lamin B receptor (LBR) [53] which is also a component of the multimeric LBR complex. Studies have shown tight binding of HABP1 to the RS motif of LBR, when the latter is not phosphorylated and it gets dissociated upon phosphorylation of LBR by LBR kinase. Thus, the interaction of LBR with HABP1 might be responsible for interaction with components of the splicing machinery. Scientists thus, speculated the prospect of RS domain phosphorylation by either SRPK1 and /or LBR kinase to function as a regulating switch for transient docking of nuclear speckles in the nuclear envelop [100].

In addition to the role of SRSF1 as a splicing regulator in association with HABP1, its structural and post-translational modification along with its shuttling nature puts it in much wider activity in other steps of RNA processing and metabolism eg. transcription of RNA polymerase II, nuclear export of mature mRNA etc. [101]; thus, regulating different cellular processes eg. genome stability, cell viability and cell cycle progression [102]. SRSF1 is found to be an essential gene since, SRSF1 null mice are embryonically lethal [99, 103] and its misregulation can result in deleterious outcomes. SRSF1 can autoregulate itself by controlling splicing of its own transcripts and ensures against overexpression by inducing the expression of PTC containing splice isoforms, which are targeted to NMD (nonsense-mediated decay). At the translational level, it is regulated by increased association of its mRNA to monosomes instead of polysomes, thereby decreasing the translation efficiency [104]. In spite of the various controlling mechanisms, SRSF1 has a propensity for overexpression due to its chromosomal localization. SRSF1 gene is located at the locus 17q23 which is frequently amplified in certain tumors and correleated with poor prognosis [105] indicating it to be a potent proto-oncogene [106]. Several reports have associated its overexpression with transformation, increased cellular proliferation and inhibition of apoptosis; suggesting its oncogenic behavior [107, 108]. Increasing evidence suggests the importance of alternatively spliced variants of tumor suppressors and oncogenes in cancer pathophysiology. SRSF1 and other splicing factors like heterogeneous nuclear ribonucleoproteins (hnRNPs) are found to be overexpressed in clinical samples and can be correlated with patient prognosis. SF2/ASF/SRSF1 is being reported as a proto-oncogene [106] which can result in spliced variants of oncoproteins. SRSF1 has been observed to be an important contributor to breast and lung cancer progression while, hnRNPA2B1 overactivity is associated with glioblastomas [108, 109]. HABP1, one of its binding partners considered to be a splicing factor regulator and together with the observations of upregulation of both HABP1 and SF2/ASF/SRSF1 in various tumors indicates its association in cancer progression at the transcript level.

**Figure 1:**
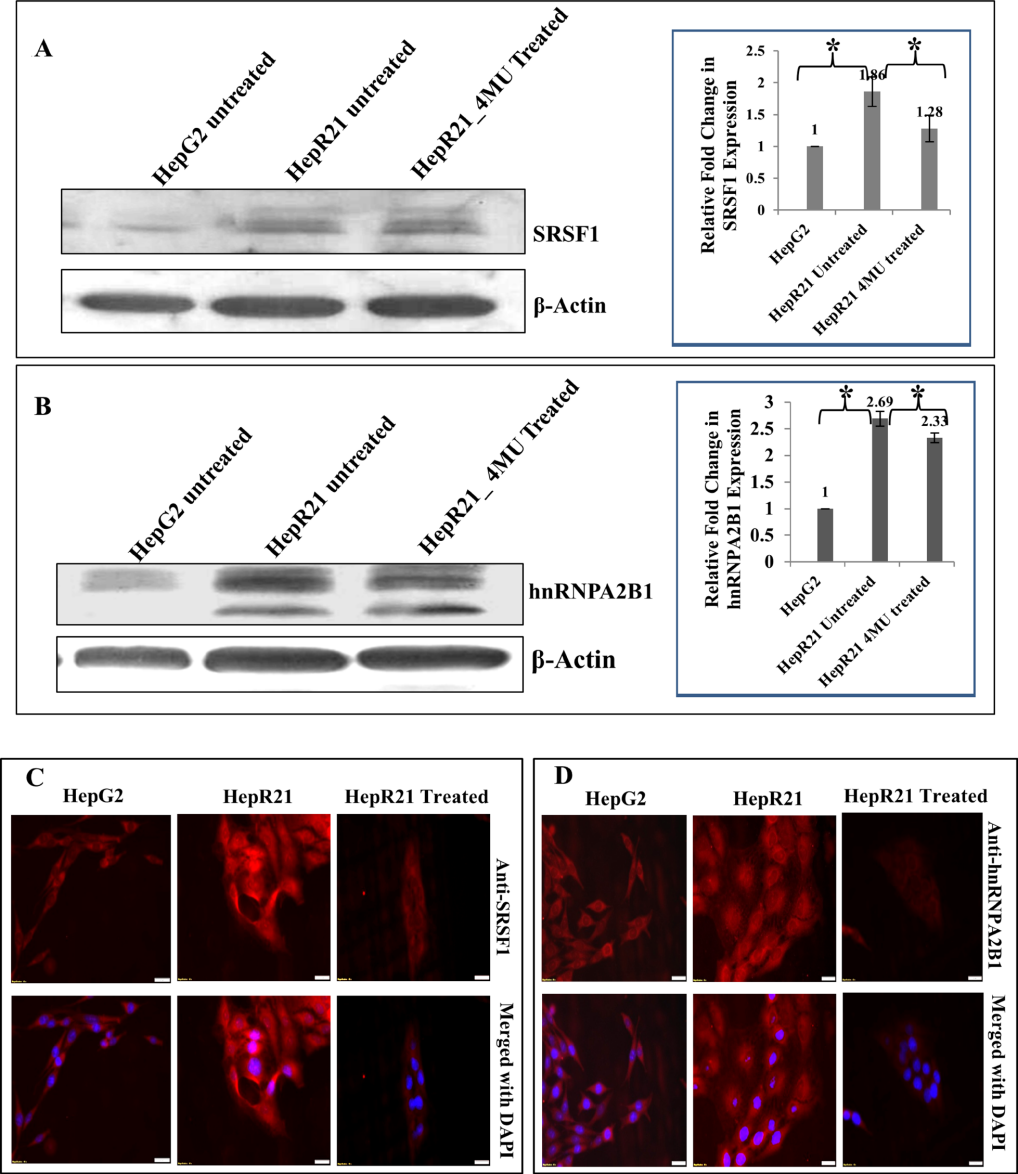
Significantly upregulated expression of the oncoproteins SRSF1 and hnRNPA2B1 in HABP1 overexpressing hepatocarcinoma cell line compared to parent cell line HepG2 Highly augmented expression of SRSF1 by ~ 1.86 folds and hnRNPA2B1 by ~ 2.66 folds was observed in HepR21 cells compared to HepG2 cells, upon immunoblotting with anti-SRSF1 and anti-hnRNPA2B1 (**A** and **B**). Fold changes were calculated after normalization with β-actin expression, using ImageJ and expressed as mean ± standard deviation (SD) of observations in triplicate (*n* = 3). Statistical analysis of significance was done by Single factor one-way ANOVA (^*^p < 0.05). This is in concurrence with the increased tumor potency in these cells compared to HepG2. While HAS inhibition through 4-MU treatment in the HepR21 cells led to downregulation of both the proteins (**A** and **B**). Further, immuno-fluorescence analysis for SRSF1 and hnRNPA2B1 in both HepG2 and HepR21 cells also revealed a prominently elevated expression of the proteins in HepR21 cells compared to HepG2 cells and a similar downregulation upon 4-MU treatment in HepR21 cells (**C** and **D**). Scale bar represents 10 μm.

Thus, armed with the knowledge of oncogenic splicing factors such as SRSF1 and hnRNPs being overexpressed in various cancers with constitutively active growth survival signals [110] and the reported co-purification and correlation with HABP1/p32 [24] we embarked upon discerning the regulatory role of SRSF1 in hepatocarcinoma cell lines HepG2 and HepG2 cell line stably overexpressing HABP1 (HepR21). HepR21 generated in our laboratory, has already been reported to have higher tumorigenic potential than its parental counterpart, HepG2 [111]. The expression profile of SRSF1 along with another splicing factor associated oncogenic protein, hnRNPA2B1 was checked in the two cell lines HepG2 and HepR21 and also in the anti-carcinogenic drug (4-MU) treated HepR21 cells. Highly augmented expression of SRSF1 by ~ 1.86 folds (Figure 1A) and hnRNPA2B1 by ~ 2.66 folds (Figure 1B) was noted in HepR21 cells which are in concurrence with the increased tumor potency in these cells compared to HepG2. While HAS inhibition through 4-MU treatment in the HepR21 cells led to downregulation of both the proteins (Figure 1A and 1B). Further, immuno-fluorescence analysis for SF2 and hnRNPA2B1 in both HepG2 and HepR21 cells also revealed a prominently elevated expression of the proteins in HepR21 cells compared to HepG2 cells and a similar downregulation upon 4-MU treatment (Figure 1C and Figure 1D, respectively).

**Figure 2:**
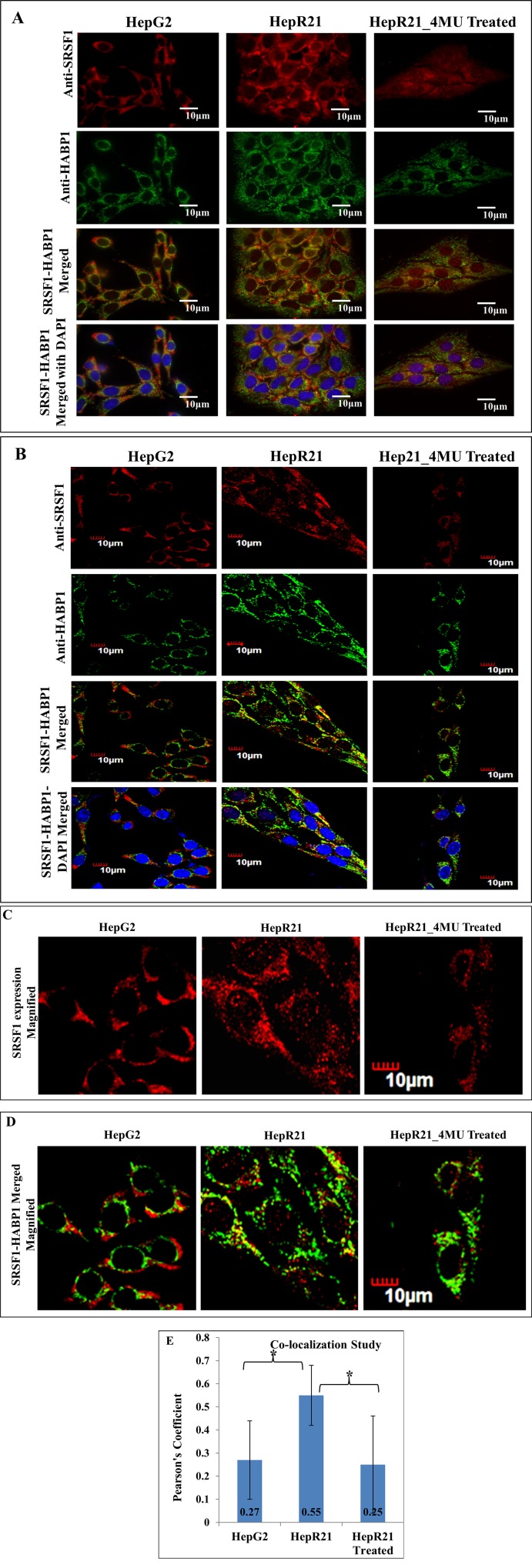
Increased co-localization of SRSF1 and HABP1 in HepR21 cells compared to HepG2 and 4-MU treated HepR21- HepG2, HepR21 and HepR21 cells treated with 4MU were immuno-stained with anti-SRSF1 and anti-HABP1 and further with Alexa Fluor 546 (for SRSF1) and 488 (for HABP1). The nuclei were stained with DAPI. All the samples were visualized initially under a fluorescence microscope (Carl Zeiss Axiovert 40 CFL) and then Confocal images (Olympus Fluoview FV1000) were also taken with the same set of samples. The increased expression of both SRSF1 and HABP1 is clearly evident in HepR21 compared to HepG2 and HepR21 treated with 4-MU (**A** and **B**). A magnified view of the confocal images of SRSF1 expression shows nuclear localization of SRSF1 in the form of speckles in the HepR21 cells (**C**), which is missing in HepG2 and HepR21 treated cells. Confocal imaging also revealed significantly increased number of co-localized points (**D**) with an average Pearson's Coefficient value of 0.55 in HepR21 cells compared to both HepG2 (0.27) and 4-MU treated HepR21 (0.25) cells (**E**). Values are represented as mean ± standard deviation (SD). For statistical analysis of significance unpaired t-test was performed using Graphpad software, where *N* = 20 and ^*^p < 0.005.

Subsequently, co-localization study between SRSF1 and HABP1 was performed and both fluorescence and confocal images corroborated the expected elevated expression level of SRSF1 and HABP1 in HepR21 cells (Figure 2A and 2B). Confocal images also revealed a significant amount of nuclear localization of SRSF1 in the form of speckles in HepR21 cells (Figure 2C). From the fluorescence images it seemed that there are positively co-localized points in HepR21, which was validated through confocal imaging and use of the Olympus Fluoview FV 1000 software. Significantly increased number of co-localized points (Figure 2D) with an average Pearson's Coefficient value of 0.55 in HepR21 cells compared to both HepG2 (0.27) and 4-MU treated HepR21 (0.25) cells (Figure 2E) was observed, which indicated positive co-localization in HepR21 cells.

It is of immense significance that SF2/ASF/SRSF1 activates mTORC1 pathway leading to proliferation of cells [112]. mTORC1 pathway regulates cell survivability by controlling many nitrogen source utilization genes. It is a known fact that activation of the mTORC1 pathway downregulates the autophagic machinery. Hence, the upregulated expression of SF2/SRSF1 can be considered to be one of the factors responsible for decreased autophagic vacuolation observed earlier in HepR21 cells [113]. Mutation in several constituents of the mTORC1 pathway can give rise to oncogenes, thereby promoting the pathway (i.e. PI3K, AKT) or inactivate tumor suppressors like PTEN which usually inhibit the pathway [114, 115]. This information supports our earlier findings showing overexpression of AKT [111] and downregulated expression of PTEN [113] in HepR21 cells, which are reported here to be having an enhanced expression of SRSF1. Moreover, numerous SRSF1 regulated alternatively spliced pro-oncogenic proteins have been identified in the promotion of tumorigenesis [99]. Out of those, it is imperative to mention here the increased exon v9 included spliced product of CD44, intron 4 retained variety of cell cycle regulatory protein, cyclinD1 (CCND1); and enhanced translation of β-catenin through mTOR activation by SRSF1 [99]. In fact, we have already reported that overexpression of HABP1/p32/gC1qR] in HepG2 Cells leads to increased HA synthesis and cell proliferation by up-regulation of Cyclin D1 in AKT-dependent Pathway [111]. There, we have also revealed elevated levels of two forms of CD44 along with increased expression of HA-CD44 mediated downstream effectors like cyclinD1 and β-catenin in HepR21 cells compared to HepG2, having lower tumor potency than HepR21 [111]. Here, nuclear localization of SRSF1 has been observed in the form of speckles. It's reported that hypophosphorylated forms of SRSF1 accumulates in nuclear speckles, where it is hyperphosphorylated by the nuclear Clk/Sty kinases and moves on to active transcription sites [99]. There is a massive difference in the splicing profile of cancer cells from that of the non-transformed cells, implicating the significance of alternate splicing in tumorigenesis and for therapeutic intervention. We have to emphasize on the fact that SRSF1 regulates not only constitutive splicing but also alternate splicing. Hence, the increased expression of HABP1 and SRSF1 in HepR21 cells, their co-localization and the nuclear localization of SRSF1 might have a role in the generation of alternatively spliced variants of certain genes and increased tumor potency in this cell line.

### Evidence of involvement of HABP1 in cancer

The knowledge of the diverse subcellular localization of HABP1 and its regulation on mitochondrial functions e.g. autophagy and apoptosis as well as splicing reactions imply its involvement in other cellular activities related to cancer progression. It is imperative to mention that; tumor microenvironment is the driving force regulating malignancy or invasiveness. Hyaluronan (HA), an ECM component and confirmed tumor biomarker is found to be enriched in the pericellular matrix or tumor stroma [116] playing a crucial role in the process of tissue remodeling by interplaying with its binding partners or “hyaladherins”. Transforming growth factor b (TGFβ) induced HA synthesis via upregulation of hyaluronan synthase 2 (HAS2) potentiates epithelial- mesenchymal transition (EMT) assisting in the migration and metastasis of cancer cells [117, 118].

Even immunological evasion is reported with the interaction of gC1qR. Tumor development is a multistage disease categorized broadly into four stages: tumor initiation, promotion, malignant conversion and progression. Long back, *Gupta and Datta* (1991) [10] and *Deb and Datta* (1996) [22] have shown that HABP1 (then referred to as hyaluronectin) interacts with HA and plays a major role in tumor cell adhesion and its secretory nature has been established on its detection in the serum free medium of macrophage tumor cell line. Inhibition of solid tumor formation was demonstrated by the treatment of macrophage cell line with the antibody raised against HABP1 [10]. A differential expression of HABP1 upon induction of epidermal carcinoma by benzo[α]pyrene (B[α]P) exposure has been observed in experimental animals [119]. Initiation is characterized by accumulation of HABP1 in inflammatory subsquamous tissue and with progression, overexpression of the protein in papillomatic and acanthotic tissue has been observed. Overexpression of HABP1 within metastatic islands characterizes the onset of metastasis while the protein disappears from the surrounding mass gradually. This observation is in accordance with features of tumor cell migration; as during metastasis, hyaluronidase degrades polymeric HA, creating spaces for migration and there is loss of interaction between its receptors and interacting proteins and their eventual downregulation [119]. HABP1 has also been reported to interact with MT1-MMP, known for having a crucial role in tumor invasion and considered as one of the effectors of metastasis. MT1-MMP degrades HABP1 by cleaving HABP1 at Gly^79^ Gln^80^ resulting in breakage of connection between structurally disordered *β*3 and *β*4 strands, thereby disrupting the HA-HABP1 interaction [39, 50]. This leads to the promotion of invasion by modifying the tissue organization. It has been suggested that this interaction might result in the disappearance of HABP1 from surrounding metastatic islands [119, 120]. Almost simultaneously, using combinatorial IgG libraries with phage display, preferential expression of gC1qR/HABP1/p32 has been reported in various adenocarcinomas like thyroid, colon, pancreatic, gastric, esophageal and lung, compared to their nonmalignant histologic counterparts [121]. Later on, *Fogal et al.* (2008), detected expression of p32 at the cell surface of tumor cell lines which has been verified via p32-binding antibodies and peptides [49]. In the tumor xenografts, p32 has been observed to be primarily localized in hypoxic/nutrient-deprived regions. In addition to tumor cells, a tumor-associated macrophage/myeloid cell subpopulation closely linked to tumor lymphatics also expresses high levels of p32. HABP1/p32 has also been identified by *Fogal* and group to be a novel receptor of the tumor homing peptide, Lyp-1, a cyclic nanopeptide that specifically recognizes lymphatic vessels in certain tumors that may be engaged in the spread of solid tumors. The protein, p32 is uniquely expressed in tumor cells, tumor lymphatics and tumor-associated macrophages/myeloid cells; which makes it a potential target for the diagnosis and treatment of cancer [49]. Further, it has been observed that upon knocking down HABP1/p32 from human cancer cells the metabolism shifts towards glycolysis from oxidative phosphorylation (OXPHOS), and the knockdowns are found to be less tumorigenic *in vivo*. The tumorigenicity can be reverted using wild type HABP1 [122, 123]. It is a universally or commonly accepted perception that, increased glycolysis under aerobic condition known as Warburg effect, enhances tumor growth.

The observation by *Fogal et al.* (2010), apparently contradicts the universally known fact of Warburg effect, since their observation of enhanced glycolysis upon knockdown of HABP1, surprisingly, led to reduced tumorigenicity, instead of augmenting tumor growth [122]. Apart from generating energy via respiration, the organelle mitochondria is crucial for the survival of cancer cells due to its participation in several other fundamental pathways e.g. pyrimidine synthesis, lipid synthesis and few others. In this connection, interplay between metabolism of glucose and the recycling of lactate which require oxidative phosphorylation is vital for tumor progression. Here it is important to mention the oncogene, Myc-induced metabolic reprogramming. Oncogenic Myc regulates glycolysis, mitochondrial biogenesis, and glutamine metabolism. Myc also induces the transcription of HABP1 and its increased expression leads to Myc-induced stimulation of glutaminolysis and glutamine addiction in brain tumors. Utilization of glutamine as energy source generates the catabolized product and TCA cycle intermediate, α-ketoglutarate enabling the tumor cells to sustain the TCA cycle activity, termed as ‘anaplerosis’. Disruption of HABP1 shows the reduced aggressiveness of tumor and the blockage of this pathway [66, 124, 125].

Keeping in mind the dynamics of differential subcellular localization of HABP1 in different cell types; we also demonstrated the association of cell surface HABP1 with cellular proliferation. Enhanced HA levels in tumor cells and accumulation of HA in tumor stroma can be correlated with cellular proliferation and the tumorigenic potential of cells. Overexpression of HABP1 in ROS insensitive hepatocellular carcinoma cell line, HepG2 induces cell surface localization of HABP1, enhanced level of endogenous HA as well as HA cable formation concomitant with cellular proliferation rate. The accumulation of HA and cell surface cable formation is reflected from the increased HA synthesis by HAS-2. HA-mediated cellular proliferation is observed with activation of MAP kinase and AKT-mediated cell survival pathway with upregulation of downstream affectors e.g. Ras, β-catenin and cyclin D1 [111]. Presumably, the surface localization of HABP1 in this stable transformant (HepR21) plays a significant part in the higher tumorigenic potential; evident from the increased survival rate in low serum condition, a shift towards anchorage independent growth and enhanced cell adhesion. This work has been further translated into 3D, using silk fibroin based three dimensional culture system, and the increased tumor potency of HepR21 has been confirmed [126]. The increased tumor potency is correlated with inhibition of autophagic vacuole formation, low serum requirement, increased cell adhesion, downregulated expression of tumor suppressor, PTEN. Hyaluronan synthase (HAS) inhibitor and the anti-carcinogenic drug, 4-MU reverts these changes with decreased tumor potency and elevated expression of tumor suppressor protein, PTEN, Beclin1 and increased autophagic vacuole formation [113]. It is also very imperative to mention here about our present observation, on the correlation of tumor potency of HepG2 cells by overexpressing HABP1 with the upregulation of oncogenic splicing factors SRSF1 and hnRNPA2B1. Along with the increased level of SRSF1 (with which HABP1/p32 was initially purified), colocalization with HABP1 and localization of SRSF1 in the nuclear speckles gives fuel to the proposition of the involvement of HABP1 in cancer progression.

Thus, the subcellular localization of HABP1 is the key determinant for governing the fate of the cell. It is well known that HA not only induces proliferation but also regulates tumor invasion and migration. HA has been shown to promote migration and metastasis in B16F10 melanoma cell line [127, 128]. Supplementation of purified HABP1 in the growth media of highly invasive melanoma cell line B16F10 enhances cellular migration, a hallmark of cancer, regulating anchorage-independent growth and aggressiveness of tumor cells. HABP1 has been observed to adhere to the cell surface and interact with integrin α_v_β_3_, a regulatory molecule of cell migration. HABP1-integrin interaction leading to enhanced tumorigenicity and cell migration by phosphorylation of nuclear inducing kinase (NIK) and IκBα followed by downstream signaling of translocation of p65 subunit of NFκB resulting in transcriptional up-regulation of MT1-MMP expression and finally MMP-2 activation is evident from *in vitro* and *in vivo* studies [129]. HABP1 induced cell migration in B16F10 melanoma cell line can be specifically blocked by either HABP1 antibody or treatment with GRGDSP, an integrin binding peptide and also with an anti-carcinogenic agent, curcumin [129]. HABP1 induced integrin-mediated migration and lamellipodia formation occurs through FAK phosphorylation and clustering, PI-3 kinase activation and actin filament bundle formation which is disrupted by antibody against either HABP1 or integrin and also upon treatment with wortmannin, a known PI3 kinase inhibitor (unpublished observation). In addition, cell surface HABP1 has also been reported to regulate cell migration through its involvement in lamellipodia formation in lung cancer cells [130] which also corroborates our findings. These observations demand its use as a therapeutic tool in future for controlling tumor aggressiveness.

The knocking down of HABP1 in growth factor inducing migrating cells exhibits disruption of lamellipodia formation with a concomitant decrease in FAK kinase and receptor kinase and simultaneously affects migration and tumorigenesis [130]. It has also been confirmed that cell surface HABP1 is indispensable for tumorigenesis and metastasis in nude mice. This observation is supported recently by interactome analysis, which unraveled the fact that HABP1 interacts with protein kinase Cζ (PRKCZ), a key regulator of not only cell polarity but also migration in EGF induced cancer cell chemotaxis. Using mass spectrophotometric identification strategy, the protein-protein interaction of HABP1 was studied and interactome network has been established. HABP1 reportedly mediates cancer cell chemotaxis by interacting with PRKCZ on the cell membrane and regulates membrane translocation activity by organizing cell polarity and induces actin polymerization [131]. Thus, from the accumulating evidence in literature it is apparent that; although the ubiquitous protein HABP1 is considered as mitochondrial, yet it has diverse cellular localization. This varied presence in different subcellular compartments e.g. cell surface and mitochondria or nucleus combined with its structural plasticity results in multitude of interactions with different ligands and cellular phenomena like proliferation and autophagy or apoptosis, respectively. It won't be irreverent here to mention that HABP1 apart from interacting with various cellular proteins that regulate metabolic pathways in cancer progression, it being a cell adherent WD family protein can also interact with several viral and bacterial proteins involved in pathogenic invasion. As for example, gC1qR can form a complex with another C1q binding protein calreticulin (CRT) [132]. During apoptosis, CRT is found to be present on the cell surface, while translocation of gC1qR into the nucleus or mitochondria is tantamount with induction of cell death [97, 133]. Interestingly, cytosolic CRT-gC1qR complex formation under high concentration of Ca^2+^ can prevent cell death by the inhibition of gC1qR translocation to the mitochondria. CRT- gC1qR/HABP1 complex potentiate viruses to proliferate and it can be a general mechanism that prevents apoptosis in cells and enable even viruses to proliferate [132]. Thus, blocking of HABP1 translocation to mitochondria either in virus infected or cancer cells results in significant amount of free HABP1; determining whether proliferation of cells or apoptosis will occur. Therefore, specific location of HABP1 inside the cell can govern the fate of the cell. Presently, both CRT and gC1qR/HABP1, which are highly conserved ubiquitous proteins, have been accepted as targets for viral manipulation and cancer therapy. The juncture between cell death and proliferation is significant for viral infections, since viruses generally aim to use the host machinery for their proliferation, thereby obstructing apoptosis. While anti-cancer chemotherapy intends to stimulate immunogenic cell death in rapidly growing cells. Thus, the aforementioned observation should be considered as vital for identifying treatment against invasion of cancer cells.

### Clinical relevance of HABP1: a tumor biomarker

All these reports have instigated scientists into investigating the expression of HABP1/p32/gC1qR and its association with cancer regulating processes using clinical samples. The early detection for tumor formation is the first step to initiate the treatment for this dreaded disease and successful treatment is partly determined by the stage of cancer progression. Clinical data supports the interactome analysis finding of this protein's involvement in cancer cell chemotaxis and metastasis [131]. A highly connected interacting network for HABP1 and protein kinase C, a key regulator of cell polarity has been constructed showing Pkcξ is regulated by HABP1 and modulated in EGF-induced cancer cell chemotaxis. Increased cell mobility is proposed to be induced by HABP1 by inducing actin polymerization indirectly through GSK3β activation. *Zhang et al* (2013) also demonstrated that high expression of HABP1 is associated with distant metastasis in patients with breast cancer [131]. Moreover, HABP1 silencing in cancer cells leads to decreased number of lung surface metastatic foci in mice model.

Several reports from last few years have also indicated highly induced expression of this protein in various human malignant tumors such as ovary [134], breast cancer [135], endometrial [136] etc. compared to their normal tissues. Epithelial tumors of breast, prostate, liver, lung, and colon, as well as in squamous and basal cell carcinoma of the skin showed strong histological staining for gC1qR/HABP1in comparison to tumors of mesenchymal origin thus *Dembitzer* and group (2012) considered this as a marker of benign and pathologic cell proliferation, particularly cells of epithelial origin [137]. *Chen et al* (2009), reported elevated levels of HABP1 mRNA and protein in breast cancer samples compared to normal breast cells and patients with low HABP1 mRNA level experienced significantly better survival rates. The study proposed that HABP1 level can act as a prognostic marker of breast cancer and elevated expression of HABP1 is related to metastasis and poor prognosis in patients [135], which is validated by further studies [138, 139]. Later, *Wang et al* (2015), linked the overexpression of this protein in breast cancer tissues and its level with distant metastasis and higher histological TNM stages in triple-negative breast cancer (TNBC) specimens [138]. Multivariate analysis revealed increased expression of HABP1 is associated with cisplatin resistance, increased risk for stage III/IV via reduced overall survival (OS) and progression free survival (PFS) in advanced serous ovarian cancer patients. Contrary to this, increased OS and PFS have been observed in patients with lower expression of HABP1 [140]. In epithelial ovarian cancer patients, augmented expression of HABP1 can act as a biomarker for lymph node and peritoneal metastasis [134]. Immunohistochemical, immunoblotting and statistical analysis of endometrial tissues revealed overexpression of HABP1 in endometrial cancer and benign endometrial lesion compared to the normal endometrium and HABP1 expression pattern is predicted to be an independent prognostic factor of OS and disease free survival [136]. HABP1 overexpression is critical for clinical progression of prostate cancer and is positively correlated with pathological stage and relapse of the disease. Selective knockdown of p32/HABP1 by RNA interference inhibits the growth of prostate cancer cell lines resulting from G1/S cell cycle arrest via downregulation of cyclinD1 and increased expression of p21 [141]. Even in case of gastric cancer, elevated level of HABP1 is correlated with progression and poor prognosis of the patients [142]. Gene polymorphism study in northern Chinese women with HABP1 upregulation, indicated the association of the single neucleotide polymorphism of minor allele of rs2285747 with increased risk of breast cancer [143]. All these clinical reports prove beyond any doubt about the validity of the multicompartmental HABP1/p32/gC1qR as a tumor tissue biomarker.

Currently, scientists are targeting cell surface antigens or vascular markers associated with tumor tissues for therapeutic intervention. Clinical reports and *in vivo* data have indicated, the multicompartmental HABP1/p32 to be considered as one such factor for cancer diagnostics and have proposed it to be a new target for antibody-based tumor targeting strategy. Monoclonal antibody (2.15 antibody) generated using antibody phase technology, is observed to be selectively taken up by mice grafted subcutaneously with MDA-MB-231 human breast cancers cells and its localization matched that of the cell surface p32/HABP1. Thus, it has been proposed that this antibody can be utilized as a vehicle for precise delivery of imaging or therapeutic agents to tumors [144]. This proposition can also be supported by the *in vitro* study showing, antibody neutralization of gC1qR/p32/HABP1, a key regulator of lamellipodia formation inhibits growth factor stimulated lamellipodia formation, cell migration and focal adhesion kinase activation. This antigen-antibody reaction inactivates receptor tyrosine kinases (RTKs) activation in various cancer cells A549 (lung cancer), MDA-MB-231 (breast adenocarcinoma, triple negative), MCF7 (primary breast adenocarcinoma) and HeLa (cervical carcinoma) *in vitro* and preventing even angiogenesis resulting in decreased tumorigenesis *in vivo* [145].

### HABP1 as a therapeutic tool

Today scientists are toiling towards developing tumor-targeted nanosystems which promise to be an effective therapeutic tool against cancer with decreased drug-induced systemic toxicity. p32/HABP1 which is expressed on the surface of activated or angiogenic endothelial cells, acts as a receptor and internalizes a nanoparticle drug, CGKRK nanoworms (NWs); consisting of elongated iron oxide coated with chimeric peptide linked via polyethelene glycol. A part of the peptide is composed of tumor-vasculature specific homing element, CGKRK while the other serving as the drug, is a membrane-perturbing proapoptotic D-amino peptide, _D_[KLAKLAK]_2_. The homing peptide directs the proapoptotic peptide to the mitochondria in target cells and has been found to be effective in orthotopic glioblastoma and breast cancer models in BALB/c nude mice [146, 147]. Earlier, HABP1 has been already mentioned to act as a receptor for the tumor homing, anti-cancerous, cyclic nonapeptide, Lyp-1 (CGNKRTRGC), which specifically recognizes an epitope in tumor-associated macrophage myeloid cells and tumor lymphatics [148]. Recently, Lyp-1 based Lyp-1-anti-DTPA bispecific antibody complex (Lyp-1-bsAbCx) and Lyp-1-doxorubincin (Dox-Lyp-1) drug conjugates and others have been generated for *in vitro* analysis of cytotoxicity in MDA-MB-231 breast cancer cells and Dox-Lyp-1 conjugates have been found to be more efficient than free doxorubicin [149]. Targeted delivery of Lyp-1 through parenteral administration was challenged as it is susceptible to degradation by blood and tissue proteases. Hence, an improved Lyp-1-mimicking peptide (TT1, CKRGARSTC) has been developed. This peptide was further used to analyze compounds having an affinity towards p32/HABP1 using fluorescence polarized-based high throughput screening and one such compound (4014008) homed to blood vessels of MCF10Ca1A breast tumor xenografts expressing p32 on cell surface [150]. Consequently, after screening a panel p32 binding peptides, a peptide designated as linear TT1 (Lin TT1) having the sequence AKRGARSTA, has been identified as the most potent tumor homing and penetrating nanosystem. The LinTT1 nanosystem has a lower affinity for p32/HABP1 resulting in lower hindrance in tissue penetration by overcoming the “binding site barrier”. LinTT1 nanosystem displayed improved tumor penetration and increased efficacy in suppressing breast tumor growth *in vivo* [151]. The efficacy of the linTT1 nanosystem has also been tested on peritoneal carcinomas expressing HABP1/p32 *in vivo*, which showed robust homing and penetration into malignant lesions compared to control tissues. Enhanced tumor selectivity and anti-tumor efficacy has been observed upon linTT1 nanoworms treatment supported by significant reduction in the peritoneal tumor weight and decline in the number of metastatic tumor nodules [152].

## CONCLUSION AND FUTURE PERSPECTIVES

Hyaluronan-binding protein 1 synonymous as HABP1/p32/gC1qR, located on human chromosome17p13.3 is a multiligand, multicompartmental and multi-functional protein. This protein has been reported to regulate diverse cellular functions such as adhesion, cellular growth, immunity, migration, apoptosis and last but not the least the processes involved in cancer progression. Alteration in the level of HABP1 has also been observed to influence cancer cell metabolism. Knocking down of HABP1 expression shifts the metabolism from oxidative phosphorylation to glycolysis which can be reverted back on retaining the higher expression of HABP1 in these cells. Several clinical reports have proved beyond any doubt the augmented expression of HABP1/p32/gC1qR in serum and tissues of patient samples, suggesting its probable use as a diagnostic and prognostic marker.

One model by which HABP1/p32/gC1qR probably achieves this functional diversity is by interacting with myriad cellular proteins via different structural components. For example, it has affinity with hyaluronan (K_d_ 1 × 10^−9^M), with the binding motif ^119^KLVRKVAGEK^128^, whereas it binds to gC1q [K_d_ (50–100) × 10^−9^M] at the N-terminal sequence under normal physiological conditions. An alternate model might invoke its ability to modulate gene expression of distinct proteins involved in diverse cellular processes. Interestingly HABP1/gC1qR/p32 was co-purified with the splicing factor SF2/SRSF1 and has been well accepted as a modulator of splicing mechanism. Moreover, the functionality of the oncogene, SRSF1 in regulating tumor aggressiveness is of significance.

Although the importance of HABP1/p32/gC1qR involved in regulating diverse cellular functions has been well recognized, only a few novel peptides and nano-particles have been attempted as a therapeutic approach. In future, deciphering the molecular partners that allow HABP1/p32/gC1qR to control each specific pathway independently, will immensely aid in designing of drugs that abolish a specific ligand mediated pathway. Further search should be continued for identifying new p32/HABP1/gC1qR affinity ligands for tumor- targeted drug delivery with increased therapeutic index and reducing drug burden in healthy tissues.
